# Do people with dementia and carers get what they need? Barriers in social care and carers needs assessments

**DOI:** 10.1177/14713012241237673

**Published:** 2024-03-01

**Authors:** Clarissa Giebel, James Watson, Julie Dickinson, Mark Gabbay, Kath Halpin, Andrew Harding, Caroline Swarbrick

**Affiliations:** Department of Primary Care & Mental Health, 4591 University of Liverpool, Liverpool, UK; NIHR Applied Research Collaborations North West Coast, Liverpool, UK; Department of Primary Care & Mental Health, 4591 University of Liverpool, Liverpool, UK; NIHR Applied Research Collaborations North West Coast, Liverpool, UK; Department of Primary Care & Mental Health, 4591 University of Liverpool, Liverpool, UK; NIHR Applied Research Collaborations North West Coast, Liverpool, UK; NIHR Applied Research Collaborations North West Coast, Liverpool, UK; Division of Health Research, 151268 Lancaster University, Lancaster, UK

**Keywords:** dementia, social care, needs assessments, inequalities

## Abstract

**Background:**

People with dementia and unpaid carers need to go through a social care or carers needs assessment to access and receive subsidised or fully-funded social care. With no previous evidence, this qualitative study aimed to provide insights into the access to, experiences of receiving and conducting social care or carers needs assessments, and access to social care.

**Methods:**

Unpaid carers of people with dementia and professionals conducting social care or carers needs assessment living or working in England were interviewed remotely about their experiences between April and August 2023. Topic guides were co-produced with two unpaid carers, and both were supported to code anonymised transcripts. Thematic analysis was used to analyse the data.

**Findings:**

Twenty-seven unpaid carers (*n* = 21) and professionals (*n* = 6) participated. Four themes were generated: (1) Issues with accessing needs assessments, not the process; (2) Knowledge of needs assessments and the health and social care system; (3) Expectations of unpaid carers; and (4) Post-assessment unmet needs. The most prominent barriers unpaid carers and their relatives with dementia encountered were awareness of and access to needs assessment. Unpaid carers were mostly unaware of the existence and entitlement to a needs assessment, and sometimes realised they had participated in one without their knowledge. Professionals described the pressures on their time and the lack of financial resources within services.

**Conclusions:**

To facilitate improved access to dementia care and support for carers, the pathway to accessing needs assessments needs to be clearer, with better integration and communication between health and social care.

## Introduction

With over 900,000 people living with dementia in the UK, and over 700,000 unpaid carers supporting someone with dementia (Alzheimer’s Society, 2021; Wittenberg et al., 2019), providing suitable care and support to both is crucial.

Social care for dementia in the UK includes both community- and long-term-care settings, such as paid home care, support groups, day care, respite care, and care homes. Social care is vital to support the needs of people living with dementia and unpaid carers to live well and as independently as possible, and support their mental health, as research at the height of the COVID-19 pandemic has shown (Giebel, Lord et al., 2021). Day care centres for example provide spaces to engage in meaningful activities and strengthen social connections (Rokstad et al., 2019). Accessing social care is means tested, and people living with dementia and unpaid carers should receive a social care needs assessment (SCNA) or carers needs assessment (CNA) when they are in need of support. People living with dementia and unpaid carers need to approach the local authority directly to request an assessment, which is conducted by local authorities and social workers. Following the assessment, a financial needs assessment is undertaken to determine to what extent those being assessed are able to afford the care needed. In situations where a person may require an enhanced level of care, but is unable to fund their care independently, local authorities are required to provide financial support. However, given well-publicised financial constraints, it is not guaranteed that local authorities will be able to fully fund thus resulting in unmet support needs.

Individual or household finances can be a barrier in accessing post-diagnostic dementia care. Nationally and globally, people living with dementia and their unpaid carers are often found to struggle getting the support they need (Caprioli et al., 2023; Giebel et al., 2021b, 2021a, 2023a). Geographical location (rural, urban, postcode) and living situation (alone or with a carer), age, gender, ethnic background, education, health and digital literacy, as well as dementia subtype are barriers to accessing and benefiting from the right support. Therefore, accessing a needs assessment from the local council is a key facilitator in accessing some forms of support and an urgent step to take for people with dementia and their carers.

While SCNA are the gateway to receiving social care support in England, to date, there is no published evidence on the experiences of receiving or conducting a SCNA or CNA for people living with dementia and unpaid carers of people living with dementia. There is some evidence reporting on the research instruments used to assess carers’ needs, such as the Carers’ Needs Assessment for Dementia (CNA-D) (Wancata et al., 2005). There is also wider evidence on unmet needs of unpaid carers for people living with dementia, including needs related to the carer as an individual, in managing the role of the carer, in delivering care, and the level and variety of dementia knowledge (Holt Clemmensen et al., 2021). Schoelzel-Dorenbos et al. (2010) explored how existing research measures of needs assessments, such as the CNA-D and the Camberwell Assessment of Needs for the Elderly (Reynolds et al., 2000), overlap with health-related quality of life and have informed a new Hierarchy Model of Needs in Dementia. Within this theoretical framework, self-actualisation and esteem needs at the top of the needs pyramid are underpinned by more basic needs of biological and physiological needs, safety needs, and belongingness and love. Specifically, the Camberwell Needs Assessment for the Elderly (Reynolds et al., 2000; Walters et al., 2000) focuses on health and physical needs, with integration of some social issues. Whilst these tools provide a suitable assessment for health and physical needs for older adults, the actual process of accessing and going through the assessment process, as well as the subsequent impact on access to dementia care has not been evidenced to date. This is a key gap in the evidence base with significant potential for impacting practice and service provision.

Without any evidence to date, the aims of this qualitative study were twofold: First, to explore the experiences of people living with dementia (by proxy) and carers about receiving a SCNA or CNA and the resulting impact on their access to and usage of social care and social support services; second, to explore the experiences of Local Authority staff of delivering SCNAs and CNAs and the resulting decision-making process of allocating funds to access social care and support services. With a growing demand of social care in England outstripping available resources (The Health Foundation, 2023), and the cost of living crisis resulting in people with dementia often having to prioritise paying for basic necessities (heating, rent, food) as opposed to being able to self-fund social care and support (Giebel & Heath, 2023b), this study will provide important insights for social care assessment and practice and a potential gateway for overcoming inequalities in care.

## Methods

### Participants and recruitment

Unpaid carers of people living with dementia and professionals conducting SCNA or CNA in England were eligible to participate. Unpaid carers had to be over the age of 18 and act as primary unpaid caregiver for someone with dementia. Professionals of Local Authority Adult Social Care (ASC) services, based within a Local Authority in England, involved in the delivery Social Care Needs Assessments (SCNA) or Carers’ Needs Assessments (CNA) for people with dementia or their carers were also eligible.

Unpaid carers and professionals were recruited predominantly through advertising on social media, and through convenience sampling via support services and Third Sector organisations, the Liverpool Dementia Action Alliance and the Liverpool Dementia and Ageing Research Forum email newsletters, and the NIHR Applied Research Collaboration North West Coast. In addition, to recruit professionals, we contacted Local Authority ASC teams across England via email. Participants from both population groups contacted either JW or CG via email. Potential participants were then given an electronic copy of the participant information sheet. If the potential participant was still willing to take part in the study, JW arranged a suitable date and time for a remote interview.

Ethical approval for this study was obtained through the University of Liverpool prior to study commencement [Ref ID: 12289].

### Data and data collection

Semi-structured interviews were conducted with participants remotely, via Microsoft Teams or Zoom conferencing software, between April and August 2023. Interviews lasted between 15 and 60 minutes and were audio-recorded for transcription purposes. Participant consent was taken via an online Google Forms form, which participants were required to complete prior to the interview. Interviews were conducted by JW, using topic guides co-produced by the research team, including public advisors and academic researchers. Two topic guides were created and used during interviews – one for unpaid carers and one for professionals (see Appendix I and II).

### Data analysis

Audio recordings of all interviews were transcribed verbatim by a paid University typist with considerable experience of transcribing research interviews. Each transcript was anonymised to ensure confidentiality of participants was maintained. Each transcript was read for accuracy, giving authors the opportunity to gain further understanding of the data and the experiences of participants. Data from transcripts were analysed using the 6-step model of thematic analysis (Braun & Clarke, 2022) by members of the research team, including training and support provided to both public advisers (CG, JW, KH, JD). Transcripts were read line-by-line, manually coded to identify the experiences of carers and Local Authority ASC staff in relation to receiving or delivering SCNA and, or CNA. Iterative re-reading of coded transcripts resulted in a condensed set of codes, which were discussed within the research team to deductively generate a set of themes and sub-themes from the data.

### Public involvement

Two public advisors (KH, JD) who are unpaid carers were involved in the research team. The public advisors were involved with the development of the research study, co-produced the topic guide, supported coding and theming of data, interpretation of the findings, and ensured the study was grounded in the applied, practical experiences of people with dementia and their carers. Both public advisors were reimbursed according to NIHR INVOLVE guidance for public involvement (2005), and wrote an NIHR Applied Research Collaboration North West Coast lay summary of the findings.

## Findings

### Participant demographics

Twenty-one unpaid carers, and six staff from Local Authority Adult Social Care and associated organisations from across England participated in the study. The participants who were unpaid carers for people living with dementia were predominantly from the North West of England (*n* = 14; 66.7%) and London (*n* = 6; 28.6%), female (*n*-12; 57.1%), from black minority ethnic groups (*n* = 16; 76.2%) and were caring for an older family member (*n* = 18; 85.7%).

### Qualitative findings

There were some overlapping themes from the interviews with carers for people living with dementia and staff providing needs assessments. There were four primary themes related to needs assessments and their subsequent care: (1) Issues with accessing needs assessments, not the process, (2) Knowledge of needs assessments and the health and social care system, (3) Expectations of unpaid carers, and (4) Post-assessment unmet need.

### Theme 1: Issues with accessing needs assessments, not the process

#### Accessing needs assessments, care reviews and subsequent care

Carers were positive in their reflections of the process of the care needs assessment(s), reporting these were a good opportunity to discuss needs and to be heard, while also receiving pragmatic advice and support from social workers which was appreciated. However, access to a needs assessment, and knowing what the assessment was for, were issues for carers. Carers often spoke of delays in accessing SCNA for people living with dementia. Some carers were unsure whether they had been involved in a needs assessment and, in certain cases, adamant they never had received one. One participant answered with a definitive “*No*” and followed up by asking how to access a social care needs assessment (**Unpaid carer, Participant 1**), another stating “*…hasn’t had any assessment as such*” (**Unpaid carer, participant 5**)”. Lack of follow-up was also an issue for carers. Many mentioned the quick-changing nature of dementia, symptomatology, and the impact of other chronic long-term health conditions, and thus a need for a revisit of a needs assessment. However, this was not always listened to by those delivering assessments:

“Once he’d [Husband living with dementia] had the seizures I wouldn’t even [leave him on his own]…so that became more intense plus his behaviour became more difficult he had to more or less have eyes to him because he was still physically strong and fit… I was still dealing with that but I was then found I was phoning [local NHS Provider] more regularly saying I don’t know what to do…and all they did was said they would speak to his consultant…it was more or less get on with it that’s the condition [Dementia]” **Unpaid carer, Participant 7**

A lack of periodic re-evaluation of care needs meant the responsibility was often on carers to contact their local authority or care organisations, leading to potential delays, unmet needs and exacerbation of poorer physical and mental health among people living with dementia and carers. When discussing additional health and palliative care needs due to an additional long-term condition, one carer noted that although they received support and financial help initially, *“there’s no follow-up [on the needs assessment]”* **(Unpaid Carer, Participant 15)**. They also noted issues in accessing social care support and healthcare effectively when discussing blood tests and making sure the person they cared for was taking their medications:

“why can’t you just talk to the [health and social care staff] and work something out before something [medical issue] happens…we get help and support from your Admiral Nurse…she can occasionally chase up stuff…But at the end of the day it’s us stood there holding the baton, coordinating all of this [medical appointments, medications and social care support]” **Unpaid Carer, Participant 15**

Proactive carers with knowledge of the health and social care system, and in a financial position to do so, were able to make adaptations to help support the person they cared for to live at home. One carer noted their lack of support or guidance from social care services in trying to adapt their parents’ home to account for their reduced physical mobility:

“we had a stairlift fitted in ours [own home] in fact we’ve got more contraptions in our house than the mobility shop. But it’s worth it, but nobody [from social care services] has been and advised, nobody has been and said oh yes you need to be doing this, you need to be doing that” **Unpaid Carer, Participant 5**

Staff working in social care and associated organisations discussed some variability in the delivery of needs assessment, both in timeliness and quality. One member of staff delivering needs assessments noted somebody’s dementia and health can deteriorate between diagnosis and when they are able to deliver the assessment. They mentioned that SCNA are *“an effective process if done in a timely manner”* (**Local Authority & Associated Staff, Participant 6)**, but that there are challenges in conducting them quickly:

“…the biggest challenge to the process [of SCNA] is the amount of people we need to get in touch with and the amount of [social care] workers we’ve got to do that. Sometimes, you’re with someone at a later stage of intervention [stage of dementia], and that could be through no fault of the process…family have tried very hard to support them…an earlier intervention would have been able to put in some more support mechanisms to keep them [person living with dementia] on an even keel or not deteriorate so quickly. Other times it is just purely they are in a list [for SCNA] and the time it has taken for us [to conduct their SCNA]” **Local Authority & Associated Staff, Participant 6**

#### Pressures on social care and staff

Staff delivering needs assessments spoke of the need to be pragmatic in light of the current staffing and funding shortfall in adult social care. This coincided with staff wanting to deliver a greater, more in-depth service for people living with dementia and carers, but this was not commensurate with their caseload:

“if we put a package of care in we would review in sort of 6–8 weeks to see how things are going and then if it’s settled we used to case hold then we got hit by COVID so we stopped…we were just dealing with what was coming in. We briefly went back to case holding which has now been taken away from us again, because I think just the volume of work our senior managers had to make that decision [remove case-holding]. I did prefer the case holding because you did get to know families and the person…we would review it [care needs] with them…and if it came back through or something came up they knew that they could contact and we would just go back out.” **Local Authority & Associated Staff, Participant 3**

Understaffing not only impacts consistency in interaction and engagement between the social care worker and the person living with dementia and their carer, but with social care support staff working at full capacity, people living with dementia are experiencing a lack of timely needs assessments. This subsequently impacted on the ability to access appropriate care packages:

“You can be waiting weeks, you can put a care package request out brokerage with a team that goes to all the agencies and sees if they’ve got capacity. Can be chasing it every single day but they just don’t have carers in that area.” **Local Authority & Associated Staff, Participant 2**

A change in care needs led to some people with dementia having to proactively contact services to try and get support. Many felt services should be re-evaluating their needs, and unpaid carers with greater knowledge of the health and social care system were likely to feel more comfortable to approach services. In contrast, unpaid carers with less experience or knowledge were less forthcoming, and would have benefitted from guidance:

“for me personally I feel if I had someone…talk me through this process and explain what I needed to know about and how to seek out support then I feel that would really have eased the process for me…I would recommend it for myself and people I know” **Unpaid Carer, Participant 3**

### Theme 2: Knowledge of needs assessments and the health and social care system

#### Lack of knowledge about terminology

Some carers were not aware that a needs assessment had taken place, what needs assessments were for, or what services were available to them subsequently. Somebody providing care for a family member seemed unsure whether they received a SCNA, asking the interviewer, “*can you just go through what a [care needs assessment is]?*” (**Unpaid carer, participant 4**). There was also confusion as to whether support could be provided before a crisis, or when they may be unable to provide care to a relative living with dementia. One carer stated that they carried out the entirety of the care for their relative, but also noted that they did not get in contact with social care, even when there were issues in providing care to their relative:

“I find it difficult to understand [my relative], very difficult trying to care [for them], because…she can’t really change [how she is / display of symptoms] and sometimes I don’t understand what she wants and it’s difficult because I have to spend most of my time with her […]. I have to make her understand literally everything. It’s very stressful trying to [help somebody to understand something] that is for their own benefit, when they’re not interested in doing it” **Unpaid Carer, Participant 13**

However, many people were unaware of what is available to them in terms of support, what they can expect from services, or what each service they have been in contact with is there to do. An interviewee who works in conducting social care needs assessments in dementia, discussed the fact that people may have been in contact with an array of services for a variety of reasons, and that issues in the dementia care pathway may cause issues:

“I think with the dementia pathways, it’s quite overwhelming and I don’t just mean that about our document [care needs assessment]. By the time they’ve got to us [local authority social care] they might have seen the GP, he’s done the mini mental…they may or may not have seen a psychiatrist…they might have seen a mental health nurse, they might have other health issues involved” **Local Authority & Associated Staff, Participant 3**

#### Lack of awareness of care services

Carers who were aware of the needs assessment process and of the services available following a diagnosis of dementia, discussed in greater detail their ability to access appropriate care. Carers who had less knowledge of the health and social care system, and of dementia, found greater difficulty in understanding what a social care needs assessment was for, in knowing where to go and who to talk to in order to access knowledge, services and support. Some carers spoke of not knowing what was available in terms of care support, and although they benefitted from having discussions with primary care, they could benefit from further information and discussions with other health and social care staff:

“I really don’t know if I need some support [social care]. I do reach out to individuals who could have a proper understanding and knowledge about such stuff…I do connect with his GP if I’m seeking support…for me I feel their support has been somewhat helpful, but if possible, I could still opt for something better than that” **Unpaid Carer, Participant 11**

#### Communication from and between services

Health care services, in particular General Practitioners (GPs), were both the initial gateway for carers accessing social care services and the point of knowledge for subsequent support. Both carers and LA staff noted that having a better relationship with health care services, and a reciprocal person-centred outlook on care, provided better results for people living with dementia and carers:

“So, the way the assessment is laid out is…we start off with a bit of background about the person, what’s important to them, what the people that support them think… whoever it is that’s important to them we ask them as well. We look at communication needs and so we try to get an idea of how much involvement with the assessment that they’ve had.” **Local Authority & Associated Staff, Participant 1**

However, communication was sometimes lacking from services and staff delivering assessments and subsequent support services, with changes in care need often resulting in greater unmet need, and a dementia diagnosis impacting on the support they would receive:

“I reached out to them [social care] it’s a bit of a criticism…once you’re diagnosed with Alzheimer’s Disease, it’s well that’s it…So as opposed to other disease [where he] would have probably had regular appointments to see what was happening with him. I got a care navigator but that was the social care bit, and they didn’t really interact at all [with healthcare].” **Unpaid Carer, Participant 7**

This was also discussed with staff involved in needs assessment delivery. Some stated that following on from a dementia diagnosis, services may not provide enough relevant information to people with dementia or carers. Sometimes, there is a lack of forthcoming support in understanding the process of needs assessments, in being referred to or accessing services, or what the SCNA was and what it can provide for the person with dementia:

“it’s almost like a lot of people living with dementia or unpaid carers] don’t understand that link between the memory assessment and the diagnosis [of dementia]. So, they actually don’t know what the follow up is from that, so they’ll [clinician] say alright you’ve got dementia, you’ve got Alzheimer’s say and then the doctors just say…we will refer you and someone will be in contact within 28 days… there’s a lot of assumption that if you’ve got some family involved that it’s their responsibility” **Local Authority Staff & Associated Staff, Participant 5**

### Theme 3: Expectations of unpaid carers

#### Pride in the independence of carer/care recipient dyad

Carers discussed their pride and the expectation that they should provide care to family members when they are in need. However, some mentioned the people living with dementia they cared for not wanting to rely on others, even family members, or having a mistrust or not wanting to have strangers (e.g., paid social care workers) in their house. The desire and pride some carers noted in providing care to their relative was important, and the person they cared for should be happy and not in a situation which may exacerbate anxiety or distress:

“[I] realised that his condition was very severe and he being the only family I have, the only family member I have left I had to quit my job because just to care for him…So, I took that career decision even though I knew perfectly it would affect my life, it would affect the things I had in mind to do, but I had to just do it because in life I believe one has to make sacrifices.” **Unpaid Carer, Participant 10**

#### Expectations of and impact on unpaid carers

Younger carers providing care to an older relative mentioned their need to give up employment or education, or reduce their social life in order to provide care for the family member. Some older carers providing care to a spouse discussed their lack of social life, and the physical and mental strain that providing care could have, particularly in times of crisis. Many carers felt that because they were in a position to care for their relative, that they were often left to their own devices, and were not given a great deal of support:

“…they didn’t really feel there was a care needs assessment for him because I was it and I was able to cope and it was lockdown so I didn’t have pressure to go anywhere… so everything was in the house and so I was doing everything.” **Unpaid Carer, Participant 7**

Unpaid carers provided a great deal of care, even in situations where formal support would be welcome and helpful due to carers’ physical issues, or a greater need for support in understanding how to best care for somebody given their symptomatology. Unpaid carers discussed a desire to keep care at home, and that maintaining a routine and a familiar environment as essential to quality of life. In countenance however, some of the staff providing needs assessments felt that although unpaid carers are in the best position to provide care, society and formal services should not place the expectation fully and solely on unpaid carers shoulders:

“…that expectation that as children we will take on that responsibility [caring for parent with dementia] and the fact that you should adapt your working life or your home life…and that brings with it, some emotions, some real depression around the fact if people can’t because they can’t give up work to look after…And some don’t want to do because they’re just not capable of doing it but then feel really guilty because they can’t deliver on what is expected by society I think sometimes.” **Local Authority & Associated Staff, Participant 4**

#### Interacting with services

Carers often discussed their need to be proactive from the outset in accessing needs assessment(s), subsequent services, and reviewing of care needs for people living with dementia as their condition progressed and changed over time. The need to be proactive was also discussed among carers who prior to the dementia diagnosis lacked knowledge of dementia and its symptoms. Many carers with less knowledge or experience discussed their need to access information, including asking questions of health and social care professionals, or searching on the internet for details on what to expect for themselves in the caring role. Carers with less knowledge initially discussed how much more confident they felt when they had a better idea of what dementia is, how to best support a people living with dementia, and what to expect over time. One carer noted the benefits of having spoken with social care staff about the support available, how they themselves can provide care to the person living with dementia, and what they may expect in the future as the dementia progresses:

“I actually spoke to them [social care] at some point I go to them to ask for…I see different changes in my auntie...I didn’t know this [what these changes were] at first, I tried to reach out to them [social care] to ask them how to cope, how to solve the problem. So, I still reach out to them.” **Unpaid Carer, Participant 9**

### Theme 4: Post-assessment care and unmet need

#### Quality of health and social care interactions

Staff facilitating access to social care discussed a desire to provide a quality service to people living with dementia and carers. Staff wanted to provide a greater frequency and level of care than they could in light of staffing and financial constraints within local authorities and social care provision. Unpaid carers discussed variable quality in interactions with health and social care services, but often, positive contacts with formal services were often unexpected and staff were seen to be going above and beyond in supporting them, reflecting somewhat low expectations of receiving care, or quality of care.

Although unpaid carers generally understood the resource constraints placed on formal social care services, the quality of their interactions with health and social care services was impacted. Unpaid carers also experienced negative contacts with healthcare and social care, and the person living with dementia endured the knock-on impact on their health. One carer mentioned the person they cared for had been in hospital for a few weeks after an emergency admission. With their health deteriorating in hospital, upon returning home, their condition improved:

“he went into hospital on the 23 June and I brought him home on about 15th August…he really was in a bad state in hospital. [He was] dehydrated. The team [district nurses / dementia team] came to see him 2 days later…They’d found he’s improved and maintained that improvement, you know, mentally.” **Unpaid Carer, Participant 12**

#### Financing of social care provision

A shortfall in knowledge is something carers discussed – notably the need for more focused information on both available services and the funding that comes from care needs assessments. Some carers felt that the needs assessment system was not functioning effectively, with funding only discussed after the assessment had taken place. Furthermore, self-funding resulted in a lack of further engagement from social care services:

“So, it’s not been a great experience [interacting with social care] because…they do the form [needs assessment], they say ‘these are their needs’ and then it’s like, ‘well she’s [person living with dementia] self-funding, figure it out yourself’…no recommendations on where to go [for support services].” **Unpaid carer, Participant 15**

This left some individuals feeling despondent and disheartened by the process. Some unpaid carers were not aware that care could be – at least in part – funded by local authorities. In fact, when asked about how their social care was funded, one carer seemed surprised that financial support may be available to pay for social care services for the person they cared for:

“No I did not get any information about that [potential financial support for social care from local authority]…I think I’m going to ask about it [financial support for social care services], because it would really help.” **Unpaid Carer, Participant 8**

Staff from local authorities and associated organisations felt delivering knowledge to people living with dementia and carers on services available was essential, as was being able to openly discuss the finances available, and what could be offered given limited funding. Additionally, with a lot of people self-financing social care, at least in part, limited personal or family finances were a barrier to accessing good quality, or frequent care for people living with dementia. Being in a position to finance their own social care gave carers and people living with dementia greater options, and being able to secure greater consistency of both carers and care quality:

“that’s one of the reasons I like using the [care] agency. I mean I have to pay for care…I went to a good [care] agency and I have the same person [carer] five days a week and he’s got to know my husband, he’s very good with him…and I feel very confident leaving him [husband living with dementia]” **Unpaid Carer, Participant 12**.

## Discussion

This is the first study, to our knowledge, that has explored the experiences surrounding social care needs assessments and carers needs assessments for dementia. One of the key findings was that carers and people with dementia experience barriers to accessing needs assessments in the first instance. However, those who had the opportunity to undertake a needs assessment reported no difficulties with the assessment itself.

Needs assessments are gateways to accessing suitable social care in the community or residential care, though this is dependent on the outcomes of those assessments and linked financial assessment. However, our study is the first to show that people with dementia and carers experienced noticeable barriers in accessing SCNA and CNA. Many carers were not aware that they or their relative were entitled to a needs assessment, and how this may provide subsidised support, depending on financial background, to care and support services. Without any prior evidence on SCNA in dementia, including their access, process, and outcome, to date, this study adds first insights into the under-researched but fundamental aspect of the social care system, and supports and extends previous evidence on wider access issues to social care services (Care England, 2023; Giebel, Sutcliffe et al., 2021). Whilst the SCNA system may be specific to the UK or its four nations, the lack of access to social care and support services for dementia is not unique to the UK, and is reported internationally (Stephan et al., 2018). To facilitate access, health and social care systems need to be better integrated, in order for GPs to directly pass on a person with a condition (in this case dementia) and their family carer to social care and suitable assessment practices. To date, this is often not the case and, as also evidenced in this study, many participants were left without any information and communication from health or social care professionals after a diagnosis.

The lack of access to needs assessments and understanding of the process and subsequent pathways to social care and support services left many people with dementia and their carers with unmet needs. Thus, there are inequalities in accessing SCNA, and access to social care services. Where Local Authorities were unable to fund all needs (also depending on an individual’s financial background), many people with dementia and their families had to self- or partially fund services. This was a barrier for many participants from accessing adequate and sufficient social care, with significant implications for the physical and mental health of people with dementia and their unpaid carers. With a dearth of evidence on outcomes of SCNA on subsequent care access, one recent related study explored resource allocation preferences in dementia care. Pierse et al. (2022) explored the views of people with dementia, carers, and health and social care professionals under constrained and unconstrained budget scenarios for social care services. The authors evidenced similar resource allocation across groups, in addition to clear preferences for psychosocial support services from people with dementia as opposed to care professionals. Carers prioritised respite services to a greater extent than other groups. This resonates with findings from this study in that unpaid carers often felt left without any support as carers themselves, having to provide high levels of unpaid care without any respite. This is repeatedly evidenced (Giebel, Hanna et al., 2021; Mansfield et al., 2023; Queluz et al., 2020), with present findings linking these needs to the lack of access to and understanding of needs assessments. Future research should explore how local authorities and staff allocate resources to different needs of people with dementia, while it would be also important to compare funding decision outcomes across different neurodegenerative conditions.

Evidenced outcome and care access inequalities were reflected in the pressures of staff conducting needs assessments. Specifically, social care staff could struggle conducting timely needs assessments, enabling subsequent access to care services, and delivering information to people living with dementia and unpaid carers. Existing evidence highlights the issues resulting from a social care service with capacity outstripped by demand (The Health Foundation, 2023). Social care staff interviewed in this study discussed the need to be pragmatic in care discussions, and carers noted a lack of expectation of what they could get from services. This emphasised that both sides are aware of the restrictions placed on social care services. Recently, the funding set-aside for social care from Central Government has been more than halved. With continuing financial pressures on social care leading to increasing levels of unmet needs (Rand et al., 2022), support for people with dementia and unpaid carers is likely to continue to fall short of demand (The Health Foundation, 2023), and with differential spending and prioritisation in local areas, is likely to lead to widening existing inequalities (Giebel et al., 2023a; Giebel & Heath, 2023) in care access and quality (Care England, 2023).

### Limitations

Whilst this is the first study exploring experiences surrounding SCNA in dementia with representation from both service users (carers) and staff conducting assessments, this study was subject to some limitations. First, we struggled recruiting people living with dementia, which may have been subject to our recruitment strategy of solely approaching social care and Third Sector organisations and recruiting via social media. Future avenues of recruitment would include NIHR Join Dementia Research, as well as recruiting via NHS memory clinics, to capture people with dementia shortly after diagnosis. Second, participants were ethnically diverse, with a substantially larger proportion of carers from a non-White ethnic background (76%). This overrepresentation of carers from minority ethnic backgrounds is not representative of the ethnic make-up of the general unpaid carer population. Thus, further research needs to explore experiences of people with dementia and carers from White ethnic backgrounds, whilst also ensuring equal representation of living location and other background characteristics, which may potentially affect SCNA experiences. Third, local authority staff were also difficult to recruit. Future recruitment should look into additional avenues, including via Integrated Care Boards and the Association of Directors of Adult Social Services (ADASS). Considering the small number of professionals conducting social care needs assessments and this being the first evidence of its kind, a larger study is required.

## Conclusions

Access to SCNA and CNA for dementia is a substantial barrier to accessing care after a diagnosis. Pressures on the social care system and staff conducting needs assessments, including funding decisions for accessing care services, create further barriers, leading to unpaid carers often having to provide care without formal support. In order to promote more equitable dementia care, needs assessments need to be more easily accessible, and understandable, with improved communication and working partnerships between and within the health and social care sector. These first findings on SCNA and CNA in dementia highlight how little knowledge is available on this key aspect of the care system, with future research needing to explore the views of people living with dementia. Findings can also inform quantitative research into how needs assessment approaches may lead to met/unmet needs and inequalities in different Local Authority areas.

## Appendix

### Appendix I: Topic guide for carers and people with dementia

Q1 To start us off, please tell us a bit about your dementia journey to date.

Q2 Please tell us about any experiences you have had of Social Care Needs Assessments for your/your relative’s dementia? *For carers: Please tell us about any experiences you have had of Carers Needs Assessments?*

Q3 How did you find out about Social Care Needs Assessments and Carers Needs Assessments?

Q4 How did you gain access to the assessment?

*Prompt: What were your experiences surrounding this – was it easy or difficult?*
*Prompt: What were your experiences of accessing a SCNA/CAN during the pandemic?*

Q5 Let us now focus on the actual process of the needs assessment.

*Prompt: Who conducted the assessment, and how was that for you?*
*Prompt: To what extent did it cover and address issues and circumstances to ensure your / your relative’s personal needs can be met?*
*Prompt: Did you receive a face-to-face or remote assessment?*

Q6 What was the outcome of the needs assessment?

*Prompt: To what extent have your/ your relative’s personal needs, or the key needs, been supported as a result?*
*Prompt: What’s not been covered and what if any challenges is that leaving you with?*

Q7 How has the Social Care Needs Assessment/ Carers Needs Assessment contributed to you/ your relative accessing, using, and funding social care and social support in the community

*Prompt: Day care/respite care/paid home carers/support groups.*

Q8 There are national rules about who has to pay for care and support. In which ways did the LA help you to access information and advice about paying for care or signpost you as to where to access the information?

Q9 Overall, what were your experiences of the needs assessment?

*Prompt: Is there anything that could or should have been done differently*
*Prompt: If so, how would that have improved the experience and/or outcome?*

Q10 Lastly, is there anything else you wish to share about the needs assessment – the process or outcomes or anything else related?

### Appendix II: Topic guide for professionals

Q1 To start us off, please tell us a bit about your role in relation to needs assessments for dementia.

Q2 Can you please talk us through the process?

*Prompt: What happens during a social care needs assessment/carers needs assessment?*

Q3 Can you please give examples of feedback from clients about the process, positive and negative and an overall impression?

Q4 What do you think about the process? What are your reflections about how effective it is?

Q5 What sort of information and advice do you provide, and how do you provide it?

*Prompt: Could you talk about some examples?*

Q6 Can you please outline whether and how this process helps people living with dementia/care partners access the appropriate support?

Q7 Can you tell us about any training relating to the process

*Prompt: If you received training, how useful was it?*
*Prompt: As an assessor, did/do you feel supported by the system as an assessor?*

Q8 How might the process be improved? How, why and for whom?

Q9 Lastly, is there anything else you wish to share about the needs assessment – the process or outcomes or anything else related?
